# Identification of Pre-Emptive Biosecurity Zone Areas for Highly Pathogenic Avian Influenza Based on Machine Learning-Driven Risk Analysis

**DOI:** 10.3390/ani13233728

**Published:** 2023-12-01

**Authors:** Kwang-Myung Jeon, Jinwoo Jung, Chang-Min Lee, Dae-Sung Yoo

**Affiliations:** 1 AI Convergence Technology Laboratory, Intflow Inc., Gwangju 61472, Republic of Korea; kmjeon@intflow.ai (K.-M.J.); wjdwlsdn1216@intflow.ai (J.J.); 2 Department of Veterinary Internal Medicine, Chonnam National University, Gwangju 61186, Republic of Korea; cmlee1122@jnu.ac.kr

**Keywords:** highly pathogenic avian influenza (HPAI), biosecurity zones, machine learning, risk analysis, pre-emptive depopulation

## Abstract

**Simple Summary:**

This research introduces a data-driven method for managing avian influenza in poultry farms, aiming to reduce unnecessary depopulation. By generating specific risk scores for farms, it significantly improves the accuracy of preventive measures against HPAI compared to traditional methods. Tested in Jeollanam-do, this approach reduces false positives, enhancing HPAI management’s reliability. The study suggests its potential for targeted farm monitoring, benefiting animal welfare and food security.

**Abstract:**

Over the last decade, highly pathogenic avian influenza (HPAI) has severely affected poultry production systems across the globe. In particular, massive pre-emptive depopulation of all poultry within a certain distance has raised concerns regarding animal welfare and food security. Thus, alternative approaches to reducing unnecessary depopulation, such as risk-based depopulation, are highly demanded. This paper proposes a data-driven method to generate a rule table and risk score for each farm to identify preventive measures against HPAI. To evaluate the proposed method, 105 cases of HPAI occurring in a total of 381 farms in Jeollanam-do from 2014 to 2023 were evaluated. The accuracy of preventive measure identification was assessed for each case using both the conventional culling method and the proposed data-driven method. The evaluation showed that the proposed method achieved an accuracy of 84.19%, significantly surpassing the previous 10.37%. The result was attributed to the proposed method reducing the false-positive rate by 83.61% compared with the conventional method, thereby enhancing the reliability of identification. The proposed method is expected to be utilized in selecting farms for monitoring and management of HPAI.

## 1. Introduction

Highly pathogenic avian influenza (HPAI) continues to pose a sustained threat to the global livestock industry and has significant socioeconomic impact [1]. According to reports from the World Health Organization, the virus has a very high mortality rate of approximately 50% when transmitted to humans. With increasing mutations, the risk of human infection is also on the rise. According to data from the Food and Agriculture Organization, the number of global incidents is steadily increasing and is expected to continue until 2023. This disease recurs every 2–3 years, and due to its rapid rate of spread, thorough reevaluation and improvement of existing disease control strategies are needed [2]. Despite many efforts, current control measures have proven insufficient in curbing the rapid spread of this disease.

South Korea, especially the Jeonnam region, has suffered significant damage from persistent and large-scale outbreaks of HPAI since 2003 [3]. Cases in this region account for approximately 20% of national outbreaks, and as of 2019, they have severely impacted the livestock industry, which constitutes approximately 6% of Jeollanam-do’s economic output. According to data from the Korean Animal Health Integrated System (KAHIS) of the Ministry of Agriculture, Food and Rural Affairs, the number of HPAI cases has been increasing in recent years. This has led to large-scale preventative culling, movement restrictions, and various resulting issues such as economic losses, price hikes, and fatigue in epidemic prevention [4]. Interestingly, South Korea is the only country in the world that performs preventative culling in all farms within a 3 km radius. During the avian flu epidemic in the winter of 2020/2021, approximately 13,015,000 birds were preventively culled within a 3 km radius of infected sites, accounting for approximately 70% of the total culled poultry. This excessive culling has led to various problems such as labor shortages, lack of burial sites, inadequacies in initial response measures, and ethical dilemmas between farms and government departments, provoking a wave of criticism against the government’s disease control policies.

In contrast, most developed countries focus on a preventive approach emphasizing individual farm disinfection and sanitation and avoiding preventive culling [5]. Countries such as the Netherlands and Hong Kong control avian flu by blocking transmission routes or using vaccines, approaches that are known to be economical and effective in the long term [6].

The high rate of culling and rapid spread of the disease indicates that there is a significant need for improvement in current disease response strategies [7]. Therefore, more effective and efficient response measures are urgently needed to mitigate the impact of this spread [8,9]. To this end, Korean health authorities are considering options including risk-based prevention and response systems in collaboration with industry, universities, and research institutes, as well as artificial intelligence (AI)-based risk prediction systems. Specifically, the adoption of AI and Internet of Things (IoT) technology allows for proactive responses for high-risk farms and regions, and plans are being explored for risk prediction and priority allocation of epidemic prevention resources.

This paper proposes a data-driven approach to strategically identify isolation zones in response to HPAI outbreaks [10], which not only enhances the precision of containment but also significantly counters the effects of unnecessary depopulation, improves animal welfare, and fortifies food security. We devise a heuristic rule table, rooted in expert opinions, to evaluate factors like geography, farm distribution, historical outbreak data, surrounding facilities, migratory bird habits, and weather patterns [11]. Utilizing this table, we conduct thorough risk assessments for each farm in the target area. The resulting data are then synthesized into a risk score for each farm through a sophisticated SVM-based algorithm, enabling a nuanced classification that informs HPAI prevention strategies. Our research is especially focused on the Jeonnam region, aiming to develop advanced early detection systems, tailored data-driven outbreak response protocols, and robust emergency decision-making frameworks. These initiatives are designed to minimize HPAI transmission risks, mitigate the repercussions of excessive culling, and maintain the stability of the agricultural sector’s contribution to the food supply chain.

## 2. Materials and Methods

### 2.1. Data Preparation and Organization

The data for identifying HPAI quarantine zones are intricately organized by major categories closely related to HPAI outbreak factors and their respective sub-categories. The final structure consists of a total of 7 major categories and 13 sub-categories. Firstly, Table 1 is composed of terrain items, including sub-categories of mountain ranges [12] and proportion of river size [13]. Table 2 addresses the status around the farm, with sub-categories including proximity to roads [14], population density [13], farm density [14], farmland ratio [12], and proximity to traditional markets. Lastly, Table 3 consists of breeding information, epidemic information, weather information, epidemiological information, and ecological environmental information, with sub-categories including breeding types, distance from nearby farms subject to analysis [15], temperature [16], wind direction [16], analysis farm occurrence history, and distance from migratory bird habitats [14]. Ultimately, all major categories are termed under one large ’rule table’.

The composition of the sub-items in the rule table is divided into two main forms. The first includes items considering the correlation between the target farm and nearby farms, such as mountain ranges, distance to nearby farms, and wind direction. These three items can verify the influence of nearby farms on the target farm based on certain conditions. The other form calculates the conditions of the sub-items based on the target farm itself. Finally, scores are set for all sub-items of the rule table.

Once the overall score setting for the conditions of the sub-items in the rule table is completed, we collect raw data that correspond to the sub-items. For the collection of items such as mountain range, proportion of river size, road proximity, population density, and distance from bird arrival areas, we utilized data from [17]. Additionally, information on farm density, farmland ratio, and proximity to traditional markets was based on data from [18]. Data related to weather, such as temperature and wind direction, were collected through [19]. Finally, information related to the farm, such as types of livestock breeding, distance to nearby farms under analysis, and outbreak history of the analysis farm, was provided through the relevant agency [20].

Following the raw data collection phase, a preprocessing step is conducted to ensure that each farm’s data can be directly applied to the rule table. In this stage, the raw data are matched to each farm according to Table 1, Table 2 and Table 3 of the entire rule table, so that each farm possesses the variables and values of the sub-items. However, for sub-items derived from interrelationships, such as distance to nearby farms, temperature, wind direction, and mountain ranges, or for weather information items that change daily, distance analysis and weather data processing are carried out using latitude and longitude coordinate values for each farm variable. Through these data configuration processes, various sub-items and rules necessary for identifying HPAI quarantine zones are accurately integrated and preprocessed for each farm, ultimately preparing them for final analysis.

### 2.2. Rule-Based Scoring

To identify the HPAI quarantine zones, the final evaluation score for all farms is calculated based on the scoring rule table. This scoring rule is structured to assign points to specific items that meet certain conditions according to the rules shown in Table 1, Table 2 and Table 3.

**Figure 1:**
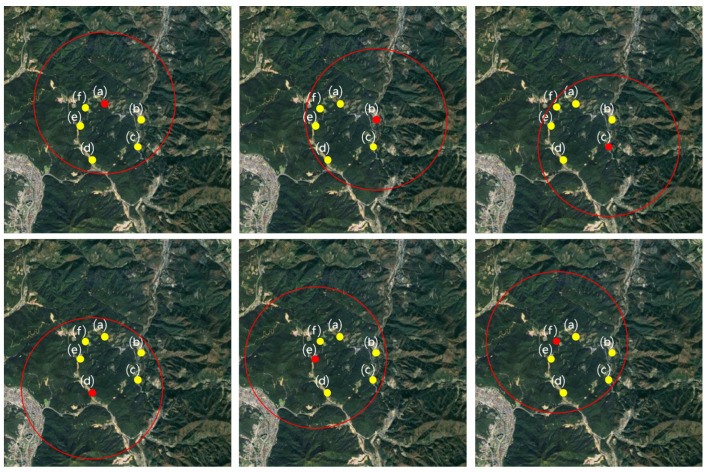
Each map contains six examples of farms subject to analysis. (a) to (f) on each map are poultry farms, the red dots on each of the six maps are the analysis target farms, the red circles are the analysis farms within a 3000 m radius, and the yellow dots are the nearby analysis farms.

The evaluation scoring method designates each chicken and duck farm across the country as an analysis target, represented by *a*. The nearby farms within a 3000 m radius from the designated analysis farm *a* are referred to as *b*. Figure 1 illustrates an example of the six processes for setting the range of these nearby farms when each farm becomes the benchmark for analysis. The evaluation score is derived by calculating the item scores according to specific rule items for these designated nearby farms. Equation (1) represents the formula to determine the single evaluation score, where $S_{a:b}$ denotes the evaluation score of farm *a* in relation to its nearby farms *b*. *m* stands for the number of rule items, and $r_{x}$ signifies the score corresponding to rule item *x*.

(1) $$S_{a:b}=\sum_{x=1}^{m}r_{x}$$

If there are no other farms within a 3000 m radius, the evaluation score for the analysis farm is calculated considering only its surrounding environmental rules.

After evaluating all the chicken and duck farms nationwide, a single evaluation score for the nearby farms within a 3000 m radius can be derived, as shown in the example of Figure 1. Table 4 depicts each example from Figure 1 in a table format and illustrates the calculation of the final evaluation score using the average value after the single evaluation scores have been derived. Equation (2) explains the method to derive the final evaluation score. Here, $FS_{a}$ is the final evaluation score of the analysis target farm *a*, *n* is the number of nearby farms within a 3000 m radius centered on the analysis target farm *a*, *k* is the number of nearby farms determined as outliers, and $S_{a:b}$ represents the single score between farm *a* and the nearby farm *b*.

(2) $$FS_{a}=\frac{1}{n-k}\sum_{b=1}^{n-k}S_{a:b}$$

### 2.3. Decision Model

SVM inherently possesses excellent generalization capabilities and is useful for building accurate and reliable classification models even with limited data [21]. It is particularly specialized for binary and multi-classification, making it highly suitable for accurately classifying whether the final evaluation score of a farm is at a dangerous level.

**Figure 2:**
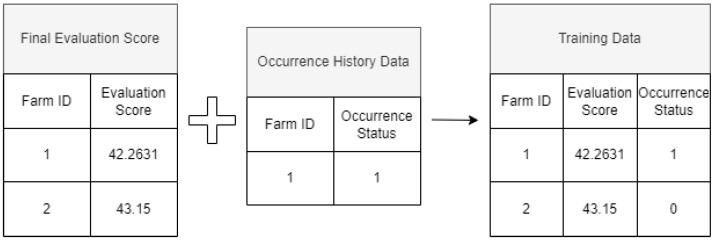
Example of the process of constructing training data by merging final evaluation score data and past occurrence history data.

**Figure 3:**
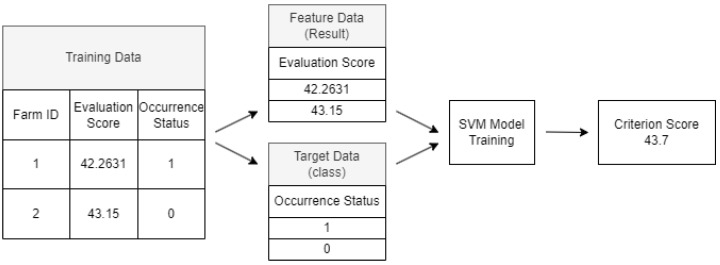
Example of the process of deriving a criterion score by dividing training data into feature data and target data.

First, during the training process, the final evaluation scores of farms nationwide that were analyzed in Section 2.2 are combined with historical occurrence data, as depicted in Figure 2. Farms with at least one past occurrence of HPAI are designated as Class 1, and farms with no such history are designated as Class 0 [22]. The completed training dataset then uses the final evaluation score just before training as the feature variable and the class information regarding occurrence history as the target variable, and training is conducted. Figure 3 illustrates the training process and the criteria for deriving the score.

**Figure 4:**
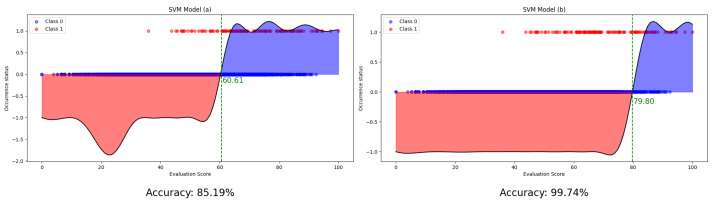
SVM model learned by setting the weights for each class to be the same (**a**) and SVM model learned by deriving the optimal weights (**b**).

However, an important challenge in this approach is the potential issue of data imbalance, given that historical data on HPAI occurrences in farms are not abundant [23]. To address this challenge, we employ a strategy involving the adjustment of class-specific weights [24]. Through iterative testing, we optimize these weights to balance the training process [25]. SVM training is subsequently performed using a Gaussian kernel to improve the model’s ability to generalize from the training data to unseen instances. The model in Figure 4a was trained by assigning equal weights to all classes, resulting in a relatively low baseline score. Consequently, the accuracy reached 85.19%. On the other hand, the Figure 4b model was trained by applying optimal weights, achieving a high accuracy of 99.74%. The accuracy difference between these two models is 14.55%, confirming that weight adjustment has a significant impact on model training.

In our upcoming experiment details, we delve into a comparative analysis illustrating the effect of these strategic weight adjustments. We designate these weights as *w*, representing the specific values assigned during the training phase. This notation aids in clearly distinguishing the contribution of each weight parameter to the model’s overall performance, highlighting the pivotal role of fine-tuning the balance between classes to enhance the prediction accuracy for HPAI occurrences [26].

Upon completion of the training phase, we establish a criterion score, which is illustrated in Figure 3, to serve as the risk threshold score for classification. Farms that have evaluation scores surpassing this criterion are labeled as “dangerous farms” that require immediate attention and potentially stringent measures. On the other hand, farms that score below this criterion are further segmented into two categories: “caution farms” and “safe farms”. This categorization is based on their average evaluation scores, allowing for a nuanced understanding of the risk levels and thereby enabling more targeted interventions.

### 2.4. Experiment Setup

In this research, we focus on HPAI issues affecting farms in the Jeollanam-do region of Korea. The temporal scope spans from 14 March 2014 to 7 April 2023, encapsulating 105 reported HPAI cases across 381 distinct farms. Designed as a bifurcated experimental inquiry, the research engages deeply with two different but related facets of the HPAI outbreak scenario.

The first experimental session adopts a micro view by examining the geographical cluster formed by each farm that has experienced an HPAI outbreak. When such a farm is identified, it becomes a point of criterion, and the surrounding farms within a 3000 m radius are closely examined. If any of these nearby farms report an HPAI case within a month from the date of the outbreak at the criterion farm, that cluster is treated as a single case for the analysis. This method allowed us to pinpoint 47 unique cases of HPAI spread in the region.

The second session shifts the lens to a macro view by analyzing the data year by year. The occurrence dates of the farms from the 47 cases of the first session are grouped by year, resulting in 8 separate case sessions corresponding to the years 2014, 2015, 2016, 2017, 2020, 2021, 2022, and 2023.

Then, the methodology remains consistent across both experimental sessions. The term “positive” is assigned to farms within the 3000 m radius of a subject farm if they also experienced an HPAI outbreak within one month of the incident at the subject farm. Those that do not meet these criteria are tagged as “negative”. Our ground-truth data are then formed based on these designations.

To evaluate various efficiencies for identifying high-risk farms, we compared four approaches using actual data. The first approach is the conventional method of culling by rule [15], which classifies all neighboring farms as ’positive’. The remaining three approaches hinge on the final evaluation scores calculated through a rule engine to classify high-risk farms. These methods, distinct from one another, depend on how the weights, designated as *w*, are adjusted. This adjustment is critical in deriving the criterion score that becomes instrumental in future risk assessments conducted via the rule engine.

The first of these, termed the *w* = 1, maintains the status quo in learning, with no weight adjustment to offset class imbalance [27], thereby not considering the ratio. This approach derives the risk criterion score based on the existing data distribution, without any regard for potential skewness between classes.

In what we have designated the *w* = 485, we take a different tack. Here, the ratio between classes 0 and 1 is meticulously adjusted to attain parity [28]. Through this method, the risk criterion score is derived by considering more nuanced factors, even if there is a pronounced imbalance within each class. This strategy allows for a more balanced view, potentially uncovering risks that a more lopsided approach might overlook.

The *w* = 8.5 represents our most refined approach. This method involves learning with the most optimized weight [29], determined through the painstaking process of fine-tuning the weight ratio. The risk criterion score in this model benefits from the most balanced and nuanced perspective, carefully honed through this optimization process.

Upon establishing these methodologies, we conducted a comprehensive comparison. We juxtaposed the actual data from individual farm sessions and annual sessions against the outcomes predicted by all four approaches.

## 3. Results

In this section, we present a comparative analysis of the experimental results from the two sessions outlined in Section 2.4 against the ground-truth data for HPAI cases in Jeollanam-do spanning from 2014 to 2023. Additionally, a confusion matrix is provided. The evaluation of the classification methods is quantified through the computation of Recall, Precision, Accuracy, and F1 scores, with the results being categorized based on the experimental method employed.

Table 5 and Table 6 in our study comprehensively compare traditional culling methods [15] with three alternative approaches (*w* = 1, *w* = 485, *w* = 8.5) based on 47 individual farm experiments. Table 5 aggregates the results, offering an average performance metric. Conversely, Table 6 classifies these results by occurrence date, presenting the annual frequency of occurrences during the experiment period in the Jeonnam region and providing a comparative analysis by year. Common to both sections, traditional methods, despite their high recovery rates, are proven markedly inefficient due to a policy of extensive culling whenever signs of trouble are detected, as evidenced by low precision and consequently low F1 scores. However, the proposed methods demonstrate respectable outcomes across all three approaches.

In detail, Table 5 shows that the traditional method exhibits a mere 10.37% accuracy. In contrast, the alternative methods, especially the *w* = 8.5 experiment, show significant improvements in both accuracy and precision. The *w* = 8.5 approach achieved 84.19% accuracy. Additionally, the value of False Positives represents the number of neighboring farms identified as False Positives among the 47 individual farm cases. This value significantly dropped from 964 in traditional methods to 158, indicating an 83.61% improvement and a substantial reduction in unnecessary culling.

Table 6 reveals that the *w* = 1 and *w* = 8.5 experimental methods yielded similar or predominantly superior results over the years. This highlights the importance of fine-tuning the Class weight in SVM training with newly emerging farms over time, and simultaneously confirms that any proposed method outperforms the traditional approach across all occurrence years.

The comparison of these two sections emphasizes the effectiveness of the proposed methods over traditional ones in minimizing collateral damage and optimizing the culling process.

## 4. Discussion

This research introduces an innovative methodology aimed at enhancing the precision of farm risk evaluations within HPAI quarantine zones. Central to this approach is the use of a diverse set of variables—geographical details, livestock facility factors, meteorological data, and epidemiological insights. This intricate blend ensures a comprehensive and reliable evaluation of farm risks, showcasing the versatility and adaptability of the model.

The study notably incorporates the SVM classification model, celebrated for its exceptional generalization capabilities, particularly in scenarios with limited datasets. A crucial aspect of the model’s effectiveness is its handling of data imbalances through class-specific weight adjustments (*w* = 1, *w* = 485, *w* = 8.5). These varying weights demonstrate the model’s adaptability and highlight the importance of weight calibration in accurately capturing the unique characteristics of each class. The varied outcomes achieved with different weights underscore the critical role these adjustments play in optimizing the model’s performance.

Additionally, the study underscores the changing patterns of HPAI transmission over the years and their impact on the model’s performance. Until 2016, HPAI transmission was primarily driven by two factors: inter-farm transmissions through fomites facilitated by local spread [30], and the movements of vehicles associated with livestock production and health [31]. A significant shift occurred post-2020, where the primary mode of transmission moved from inter-farm to contamination of the farm surroundings by wild birds, resulting in viral incursion [32].

Importantly, starting in 2018, the implementation of temporary downtime periods in domestic duck holdings also played a crucial role in altering the transmission dynamics. This intervention significantly reduced the spread of HPAI between farms and to surrounding areas, impacting the frequency and pattern of outbreaks. These distinct shifts in transmission modes—from fomite-based and vehicle-associated spread to wild bird activities, coupled with the effective downtime strategy in duck farms—have considerably influenced the performance of the risk assessment model [13].

However, the study is not without limitations. One of the primary constraints was the reliance on expert-derived rule scores, due to which there was an inherent limitation in capturing the complete complexity of HPAI transmission. Additionally, due to concerns related to personal information and other challenges, the study faced difficulties in collecting detailed data on vehicle and personnel movements. This lack of comprehensive data on inter-farm interactions limited our ability to fully understand the intricacies of farm-to-farm transmission, posing challenges in refining the model to its utmost potential.

Despite certain limitations, the unique strengths and potential applications of this approach merit attention. Distinct from conventional strategies that rely on generic and often imprecise regional risk analyses, this methodology advocates for bespoke risk evaluations at the individual farm level. Such specificity enables targeted culling measures, avoiding the pitfalls of unnecessary and indiscriminate actions. Its adaptability to a variety of disease contexts marks a notable advancement over traditional epidemiological methods, which may lag in terms of response time. By minimizing unwarranted culling through meticulous farm-specific risk assessments, this approach not only significantly enhances animal welfare but also offers notable economic advantages. This tailored strategy exemplifies the importance of precise risk management in diverse disease scenarios and aligns with broader goals of sustainable and responsible animal husbandry.

Moreover, the potential to apply this methodology across a range of disease scenarios, provided that appropriate rule sets can be developed, highlights its versatility and broad applicability. The ability to adapt this approach to various diseases, including foot-and-mouth and African swine fever, demonstrates its practicality and the far-reaching implications of this nuanced method. By offering a flexible framework adaptable to different disease outbreaks, it sets a new standard in the field of disease management, underscoring the critical role of adaptive strategies in contemporary veterinary practices and public health management.

## 5. Conclusions

This research presents a transformative approach in animal disease management, with a focus on HPAI. By integrating diverse variables and utilizing the SVM classification model for nuanced data analysis, it enhances precision in farm risk assessments. The study, while acknowledging certain limitations like the reliance on expert-derived rules and data collection challenges, demonstrates the model’s flexibility across various animal diseases, such as foot and mouth disease and African swine fever. A key outcome of this study is the potential to significantly reduce unnecessary culling by precisely identifying animals for culling through advanced technology. This represents a shift towards more intelligent, science-based control strategies, steering away from traditional methods and towards more effective and sustainable animal health management practices.

## Figures and Tables

**Table 1:** Decision rules related to geographical information for HPAI quarantine zone designation.

Highly Pathogenic Avian Influenza (HPAI) Farm Culling Criteria			Score
Terrain	Mountain range	In cases where there are mountain ranges or terrains with an altitude of 50 m or higher blocking the direct path between the farm under analysis and the nearby farm.	−10
		If the farm under analysis is located in the mountains or a mountain is within 100 m proximity.	+3
	Proportion of river size	When the proportion of national rivers or local rivers within 3 km of the farm under analysis is 3% or higher.	+5
		When the proportion of national rivers or local rivers within 3 km of the farm under analysis is between 2% and 3%.	+2
		When the proportion of national rivers or local rivers within 3 km of the farm under analysis is between 1% and 2%.	+1
		When the proportion of national rivers or local rivers within 3 km of the farm under analysis is 1% or less.	−2

**Table 2:** Decision rules related to the status around farms for designating HPAI quarantine zones.

Highly Pathogenic Avian Influenza (HPAI) Farm Culling Criteria			Score
Status around the farm	Road proximity	In cases where the distance between the farm under analysis and the surrounding road (with 2 or more lanes) is within 1 km.	+5
		In cases where the distance between the farm under analysis and the surrounding road (with 2 or more lanes) is between 1 km and 3 km.	+2
		In cases where the distance between the farm under analysis and the surrounding road (with 2 or more lanes) exceeds 3 km.	−3
	Population density	When the population density of the administrative area where the farm under analysis is located is 100 or more per 1 km${}^{2}$.	+5
		When the population density of the administrative area where the farm under analysis is located is 50 or more per 1 km${}^{2}$.	+2
		When the population density of the administrative area where the farm under analysis is located is 30 or more per 1 km${}^{2}$.	+1
		When the population density of the administrative area where the farm under analysis is located is 20 or fewer per 1 km${}^{2}$.	−2
	Farm density	When the combined number of poultry and duck farms in the administrative area of the farm under analysis is 1 or more per 1 km${}^{2}$.	+10
		When the combined number of poultry and duck farms in the administrative area of the farm under analysis is between 0.5 and 0.1 per 1 km${}^{2}$.	+5
		When the combined number of poultry and duck farms in the administrative area of the farm under analysis is between 0.3 and 0.5 per 1 km${}^{2}$.	+2
		When the combined number of poultry and duck farms in the administrative area of the farm under analysis is 0.3 or fewer per 1 km${}^{2}$.	−2
	Farmland ratio	When the proportion of farmland within 3 km of the farm under analysis is 30% or more.	+5
		When the proportion of farmland within 3 km of the farm under analysis is between 20% and 30%.	+2
		When the proportion of farmland within 3 km of the farm under analysis is between 10% and 20%.	+1
		When the proportion of farmland within 3 km of the farm under analysis is 10% or less.	−2
	Traditional market	When the distance between the analysis target farm and the market is less than 1 km.	+5
		When the distance between the analyzed farm and the market is more than 1 km and less than 2 km.	+3
		When the distance between the analysis target farm and the market is more than 2 km and less than 5 km.	+2
		When the distance between the analysis target farm and the market exceeds 5 km.	−1

**Table 3:** Decision rules related to breeding information, infectious disease information, meteorological information, epidemiological information, and ecological environment information for designating HPAI quarantine zones.

Highly Pathogenic Avian Influenza (HPAI) Farm Culling Criteria			Score
Breed	Breeding type	In the case where the farm under analysis raises breeding chickens.	+0
		In the case where the farm under analysis raises meat chickens.	+0
		In the case where the farm under analysis raises laying hens.	+5
		In the case where the farm under analysis raises breeding ducks.	+20
		In the case where the farm under analysis raises meat ducks.	+15
Epidemic information	Analyzing nearby farm distances	If the distance between the farm under analysis and the nearby farm is within 500 m.	+30
		If the distance between the farm under analysis and the nearby farm is 500 m~3 km.	+5
		If the distance between the farm under analysis and the nearby farm is 3 km~10 km.	+2
		If the distance between the farm under analysis and the nearby farm exceeds 10 km.	−5
Weather	Temperature	If the temperature on the day of analysis is below 0 ${}^{{}^{\circ}}$C.	+7
		If the temperature on the day of analysis is 0 ${}^{{}^{\circ}}$C~15 ${}^{{}^{\circ}}$C.	+5
		If the temperature on the day of analysis is 15 ${}^{{}^{\circ}}$C~20 ${}^{{}^{\circ}}$C.	+3
		If the temperature on the day of analysis is 20 ${}^{{}^{\circ}}$C~30 ${}^{{}^{\circ}}$C.	+0
		If the temperature on the day of analysis exceeds 30 ${}^{{}^{\circ}}$C.	−10
	Wind direction	If the wind blows from the nearby farm under analysis towards the farm under analysis at an average speed of 3.3 m/s or more on the day of analysis.	+5
Epidemiological history	Analysis farm occurrence history	In the case where the farm under analysis has had one occurrence of HPAI in the past 5 years.	+10
		In the case where the farm under analysis has had two occurrences of HPAI in the past 5 years.	+20
		In the case where the farm under analysis has had three occurrences of HPAI in the past 5 years.	+40
Ecological environment	Distance from migratory bird habitat	If the distance between the farm under analysis and the main migratory bird habitat is within 15 km.	+7
		If the distance between the farm under analysis and the main migratory bird habitat is 15~30 km.	+3
		If the distance between the farm under analysis and the main migratory bird habitat exceeds 30 km.	−3

**Table 4:** Final evaluation score calculated with each farm being analyzed once from (a) to (f) in Figure 1.

Farm Name	Evaluation Score	Final Evaluation Score
(a)	$S_{\left(a):(b\right)}$ = 45, $S_{\left(a):(c\right)}$ = 30, $S_{\left(a):(d\right)}$ = 48, $S_{\left(a):(e\right)}$ = 32, $S_{\left(a):(f\right)}$ = 38	$FS_{\left(a\right)}=38.6$
(b)	$S_{\left(b):(a\right)}$ = 50, $S_{\left(b):(c\right)}$ = 54, $S_{\left(b):(d\right)}$ = 55, $S_{\left(b):(e\right)}$ = 60, $S_{\left(b):(f\right)}$ = 58	$FS_{\left(b\right)}=55.4$
(c)	$S_{\left(c):(a\right)}$ = 12, $S_{\left(c):(b\right)}$ = 16, $S_{\left(c):(d\right)}$ = 18, $S_{\left(c):(e\right)}$ = 20, $S_{\left(c):(f\right)}$ = 9	$FS_{\left(c\right)}=15$
(d)	$S_{\left(d):(a\right)}$ = 50, $S_{\left(d):(b\right)}$ = 54, $S_{\left(d):(c\right)}$ = 55, $S_{\left(d):(e\right)}$ = 60, $S_{\left(d):(f\right)}$ = 58	$FS_{\left(d\right)}=55.4$
(e)	$S_{\left(e):(a\right)}$ = 88, $S_{\left(e):(b\right)}$ = 76, $S_{\left(e):(c\right)}$ = 74, $S_{\left(e):(d\right)}$ = 79, $S_{\left(e):(f\right)}$ = 85	$FS_{\left(e\right)}=80.4$
(f)	$S_{\left(f):(a\right)}$ = 33, $S_{\left(f):(b\right)}$ = 35, $S_{\left(f):(c\right)}$ = 50, $S_{\left(f):(d\right)}$ = 48, $S_{\left(f):(e\right)}$ = 39	$FS_{\left(f\right)}=41$

**Table 5:** Individual farm session comprehensive evaluation index.

Experiment Method	False Positive	Recall	Precision	Accuracy	F1 Score
Conventional Method	964	1	0.1037	0.1037	0.1817
Proposed Method (*w* = 1)	154	0.7865	0.4252	0.8408	0.5046
Proposed Method (*w* = 485)	597	0.9851	0.1805	0.4537	0.2829
Proposed Method (*w* = 8.5)	158	0.839	0.489	0.8419	0.5619

**Table 6:** Session evaluation metrics by year.

Year of Occurrence	Number of Occurrences per Year	Experiment Method	Recall	Precision	Accuracy	F1 Score
2014	8	Conventional Method	1	0.093023	0.093023	0.17
		Proposed Method (*w* = 1)	0.75	0.222222	0.732558	0.343
		Proposed Method (*w* = 485)	1	0.145455	0.453488	0.254
		Proposed Method (*w* = 8.5)	0.75	0.222222	0.732558	0.343
2015	63	Conventional Method	1	0.134043	0.134043	0.236
		Proposed Method (*w* = 1)	0.952381	0.437956	0.829787	0.6
		Proposed Method (*w* = 485)	1	0.225806	0.540426	0.368
		Proposed Method (*w* = 8.5)	0.904762	0.431818	0.82766	0.585
2016	5	Conventional Method	1	0.060241	0.060241	0.114
		Proposed Method (*w* = 1)	1	0.138889	0.626506	0.244
		Proposed Method (*w* = 485)	1	0.104167	0.481928	0.189
		Proposed Method (*w* = 8.5)	1	0.138889	0.626506	0.244
2017	5	Conventional Method	1	0.054348	0.054348	0.103
		Proposed Method (*w* = 1)	1	0.714286	0.978261	0.833
		Proposed Method (*w* = 485)	1	0.076923	0.347826	0.143
		Proposed Method (*w* = 8.5)	1	0.714286	0.978261	0.833
2020	1	Conventional Method	1	0.02381	0.02381	0.047
		Proposed Method (*w* = 1)	1	0.111111	0.809524	0.2
		Proposed Method (*w* = 485)	1	0.034483	0.333333	0.067
		Proposed Method (*w* = 8.5)	1	0.125	0.833333	0.222
2021	2	Conventional Method	1	0.057143	0.057143	0.108
		Proposed Method (*w* = 1)	1	0.4	0.914286	0.571
		Proposed Method (*w* = 485)	1	0.090909	0.428571	0.167
		Proposed Method (*w* = 8.5)	1	0.4	0.914286	0.571
2022	19	Conventional Method	1	0.097436	0.097436	0.178
		Proposed Method (*w* = 1)	0.368421	0.466667	0.897436	0.412
		Proposed Method (*w* = 485)	0.894737	0.114865	0.317949	0.204
		Proposed Method (*w* = 8.5)	0.631579	0.461538	0.892308	0.533
2023	2	Conventional Method	1	0.030303	0.030303	0.059
		Proposed Method (*w* = 1)	1	0.333333	0.939394	0.5
		Proposed Method (*w* = 485)	1	0.037037	0.212121	0.071
		Proposed Method (*w* = 8.5)	1	0.285714	0.924242	0.444

## Data Availability

The raw data concerning the farms showcased in this study are available subject to the permission of the relevant institutions.
